# Two fatal cases of melioidosis on the Thai-Myanmar border

**DOI:** 10.12688/f1000research.3-4.v2

**Published:** 2014-03-31

**Authors:** Cindy S. Chu, Stuart Winearls, Clare Ling, Miriam Beer Torchinsky, Aung Phae Phyo, Warat Haohankunnathum, Paul Turner, Vanaporn Wuthiekanun, François Nosten

**Affiliations:** 1 Shoklo Malaria Research Unit, Mahidol-Oxford Tropical Medicine Research Unit, Faculty of Tropical Medicine, Mahidol University, Mae Sot, 63110, Thailand; 2 Centre for Tropical Medicine, Nuffield Department of Medicine, University of Oxford, Oxford, OX37 7FZ, UK; 3 Department of Medicine, University of British Columbia, Vancouver, V5Z 1M9, Canada; 4 Cambodia-Oxford Medical Research Unit, Angkor Hospital for Children, Siem Reap, Cambodia; 5 Mahidol-Oxford Tropical Medicine Research Unit, Faculty of Tropical Medicine, Mahidol University, Bangkok, 10400, Thailand

## Abstract

Melioidosis is endemic in areas of Southeast Asia, however, there are no published reports from the Thai-Myanmar border. We report the first two documented cases of fatal melioidosis in this region. This is of great public health importance and highlights the need to both increase clinical awareness of melioidosis on the Thai-Myanmar border, and to assess the true burden of disease in the area through improved case detection and *Burkholderia pseudomallei* prevalence studies.

## Presentation

Melioidosis, caused by *Burkholderia pseudomallei*, is endemic in areas of Southeast Asia. Published case reports from the Thai-Myanmar border are absent and the general belief is that melioidosis does not occur in this region. Presented below are two case reports suggesting that melioidosis does in fact occur and may be under-recognized and not reported.

The first case of this series was a 17-year old female who presented with an intermittent fever for two months, more than 10% weight loss and confusion. She had been previously healthy. Her parents were farmers and the family lived on their farm, growing rice, corn and beans, in eastern Myanmar along the Thailand border. On physical examination, her temperature was 40.1°C, pulse 130 bpm, respiration 28/min, and blood pressure 80/50 mmHg. In general, she was prostrate with conjunctival pallor. Her Glasgow Coma Score was 10/15. The cardiovascular and pulmonary exams were normal aside from tachycardia and tachypnea. Abdominal examination revealed hepatosplenomegaly. There were no other significant findings on clinical exam. Evidence from a random dextrose level test (432 mg/dL) and a urine dipstick; white blood cell 1+, ketone 3+, glucose 1+; were consistent with anorexia. Repeated random dextrose levels remained elevated and the maximum result was 520 mg/dL. A malaria smear showed *Plasmodium vivax* at a low parasitemia. Because of decreased consciousness, a lumbar puncture was performed and clear cerebrospinal fluid (CSF) was obtained. The CSF cell counts were normal and no organisms were grown after 48 hours of culture. Blood cultures were also sent. The patient was resuscitated with normal saline, given ceftriaxone 1 g intravenous BID and stabilized. Treatment with chloroquine 500 mg oral daily was started for *P. vivax* malaria. Over the following two days, further laboratory results were obtained. A complete blood count (CBC) showed an elevated white blood count (WBC) at 13.5 10 ^3^/µL with a neutrophilia, hemoglobin (Hgb) 14.1 g/dL and a normal platelet count. Chest X-ray was abnormal with a left sided consolidation, cavitation on the right side and bilateral hilar lymphadenopathy. Sputum tested for acid fast bacilli was negative. Despite the addition of gentamicin 280 mg intravenous daily and appropriate supportive measures, the patient deteriorated and died within three days of hospitalization, more than eight weeks after the onset of symptoms. Admission blood culture flagged positive after one day of incubation and an identification of *B. pseudomallei* was made and communicated to the clinical staff one day after the patient’s death.

The second case was a 45-year-old male who presented with fever and weakness for two days. He also complained of chills, rigors, cough and generalized body aches. He had a history of gastric ulcer and hypertension, neither of which were currently being treated. He was a rice farmer on the eastern border of Myanmar and Thailand and had continued to work until he fell ill. He was treated with ceftriaxone 1 g intravenous daily and metronidazole 500 mg oral TID by a village health worker. At this time a malaria smear was negative and other diagnostic testing was not performed. On the sixth day of illness he had still not improved and was sent to our clinic. At initial consultation, tympanic temperature was 36°C, pulse 76 bpm, respiration 20/min, and blood pressure 100/70 mmHg. He was generally weak but ambulatory with a Glasgow Coma Score of 15/15. He had icteric sclera and pale conjunctivae. The cardiovascular, pulmonary and abdominal exams were normal. On skin examination, a 3 cm lesion that appeared like a superficial abscess was noted on the right leg. Additional history revealed that this lesion had intermittently appeared and resolved over more than two years.

During his hospitalization, the patient developed congestive heart failure and pulmonary edema that improved with initial management. A random venous dextrose level was 110 mg/dL and field hematocrit 27%. A Urine dipstick result was abnormal with protein 2+ and blood 4+. Urine sediment was normal. Dengue rapid test was NS-1 negative, IgM and IgG positive (Standard Diagnostics, Inc, Kyonggi province, Korea). A scrub typhus rapid test for total antibody was positive (Standard Diagnostics, Inc). Stool test microscopy was negative and the stool sample was noted to be of black color. His CBC showed a Hgb 8.1 g/dL, WBC 26.2 10 ^3^/µL with a neutrophilia and normal platelets. Blood culture was negative. Biochemistry was sent to the district hospital, however, the results were not available immediately. His treatment regimen included cloxacillin 1 g intravenous QID, ceftriaxone 1 g intravenous daily, doxycycline 100 mg oral BID and furosemide 40 mg oral BID. Six days after admission the biochemistry results were returned and showed markedly abnormal renal function; blood urea nitrogen (BUN) 112.5 mg% and creatinine 15.4 mg%. He was diagnosed with chronic renal failure exacerbated by dengue and scrub typhus infections. After two weeks of hospitalization, the patient’s clinical condition deteriorated and he died despite prolonged broad antibiotic coverage. A second blood culture obtained one day prior to his death flagged positive after one day of incubation, was identified as *B. pseudomallei* and results were communicated to the clinical staff one day after his death.

## Diagnosis

*B. pseudomallei* isolates from both cases were obtained using the BacT/Alert ^®^ 3D automatic blood culture system (bioMérieux, Marcy L’Etoile, France). The colonies of both isolates on blood agar after approximately 24 hours of incubation were non-haemolytic, smooth, white and domed shaped. On Gram staining, both isolates showed Gram negative rods with characteristic ‘safety pin’ appearances. The isolate from case one was oxidase positive and initially identified as *B. pseudomallei* using the API 20NE test kit (bioMérieux) which gave an 81.7% match (profile number 1056576). It was then confirmed to be *B. pseudomallei* using a highly specific *B. Pseudomallei* latex agglutination test. The *B. Pseudomallei* latex test, produced and provided by Mahidol Oxford Tropical Medicine Research Unit (MORU), is based on an anti-exopolysaccharide monoclonal antibody ^1^. The ** isolate from case two was also oxidase positive and immediately identified as *B. pseudomallei* using the same *B. Pseudomallei* latex test. Both isolates were sent to MORU for confirmation, where they were found to have typical colony morphologies on Ashdown’s agar (purple colour, metallic sheen, becoming dry and wrinkled after 48 hours), gave positive results to oxidase and latex agglutination test, L-arabinose negative, resistant to gentamicin and colistin. Susceptibility testing by disc diffusion was performed at Shoklo Malaria Research Unit (SMRU) for the first isolate and at MORU for the second ( Table 1). Two different colony types were found for case 2; one, which was susceptible to meropenem, and one with intermediate susceptibility to meropenem (bioMérieux Etest minimum inhibitory concentration of 8 μg/mL).

**Table 1.** Susceptibility profiles of *B. pseudomallei* isolates from case one (1) and case two (2i and 2ii).

Case	Gentamicin	Amoxicillin clavulanic acid	Ceftazidime	Doxycycline	Meropenem	Imipenem	Co-trimoxazole
**1**	R	S	S	NT	S	NT	S
**2i**	R	S	S	S	S	S	S
**2ii**	R	S	S	S	I	S	S

R=resistant, S=sensitive, I=intermediate, NT=not tested

## Management

Antimicrobial therapeutic options for melioidosis are limited due to the intrinsic resistance of the organism. Current guidelines recommend parenteral ceftazidime 50 mg/kg up to 2 g every 6 hours or meropenem 25 mg/kg up to 1 g every 8 hours for at least 10–14 days (longer in severe or complicated cases) followed by oral co-trimoxazole plus doxycycline (first-line) or co-amoxiclavulanate alone (second-line) for 20 weeks. In these cases, empiric ceftazidime or meropenem was not given due to the low suspicion for *B. Pseudomallei* sepsis. New data support the use of mono-therapy with oral trimethoprim-sulfamethoxazole for the oral phase of treatment ^15^. This simplifies the treatment regimen and should have a positive impact on prescribing patterns in suspected cases.

## Discussion

At the beginning of the 20 ^th^ century in Rangoon, Burma, Dr. Alfred Whitmore and his assistant Krishnaswami identified a new bacteria causing disease in humans. It was originally named *Bacillus pseudomallei* ^2, 3^ and renamed *Burkholderia pseudomallei* in 1992 ^4^. They proceeded to document over a hundred cases and the bacterium was isolated in over 5% of Rangoon’s autopsies at that time ^5^. The disease, now termed melioidosis, was named by Stanton and Fletcher in 1932 due to its clinical resemblance to glanders (an infectious disease of horses) ^6^. It can present with an array of clinical symptoms but is most commonly characterised by pneumonia, abscess formation, and severe sepsis, and is associated with a high mortality ^7^. It has become an important public health concern across Southeast Asia and northern Australia. In this region, incidence rates vary between 1.1/100000 and 412.7/100000 ^8^, and Lao PDR has recorded 400 cases between 1999 and 2010 ^9, 10^. This variability may be explained by the geographical preference of *B. pseudomallei* for specific soils ^11, 12^, population risk factors that include occupation (rice farming) ^8^ or medical conditions such as diabetes, renal failure and immunosuppressed states ^7^. Another factor in documentation of cases is the availability of laboratories for culture diagnosis ^7^. Little is known about the current disease burden in Myanmar and along its borders, the last published case from within Myanmar having been reported in 1945 ^7^. A recent serological survey of new Burmese migrants to Thailand revealed that 78% were seropositive for antibodies to *B. pseudomallei* ^13^. This high seropositivity rate is supported by Aung *et al.* who found that 2% of pus specimens obtained from abscesses from patients examined in Yangon, also known as Rangoon, Myanmar, contained organisms consistent with *B. pseudomallei* ^14^.

The first case described here was a healthy young female and her only known risk factor was environmental exposure. The elevated dextrose on admission was attributed to glycemic dysregulation rather than from new onset diabetes given the two month duration of fever and symptoms prior to hospitalization. However, a glycated hemoglobin was not performed and diabetes could not be completely ruled out. Concurrent vivax malaria infection may have caused immunosuppression enabling sepsis with *B. pseudomallei*. The second case was a male with environmental exposure as well as undiagnosed chronic renal disease. The first blood culture was negative, and concurrent infection with dengue and possibly scrub typhus (the rapid test used detected IgG, IgM and IgA as total Ab and could not differentiate acute or resolved infection) may have caused worsening immunosuppression resulting in *B. Pseudomallei* sepsis. Both cases had risk factors and concomitant infections.

In these cases, death occurred shortly after admission and the diagnosis of melioidosis was not considered until after bacteriologic results were available. Following the first case, a latex test is now performed on all oxidase positive Gram negative isolates which was not routine previously. If the clinical suspicion of melioidosis was higher, appropriate antibiotics could have been started earlier in the hospital course. In general, ceftazidime is available in Thailand and Myanmar, however, hospital care and treatment is difficult to access for the impoverished migrant populations. At SMRU field hospitals, ceftazidime was not routinely stocked, and as a consequence of these two cases, all sites with inpatient wards now carry ceftazidime.

## Conclusion

Melioidosis has not been reported to be endemic in eastern Myanmar along the Thailand border, therefore, health providers may not consider it as a diagnosis in persons with sepsis. The detection of *B. Pseudomallei* in this case series highlights the need for further epidemiologic study and case diagnosis in this region.

## Consent

Written informed consent to report these cases was obtained from the families of the patients.
