# Supplementary material for: Two fatal cases of melioidosis on the Thai-Myanmar border
Source: F1000Res. 2014 Mar 31;3:4. Originally published 2014 Jan 6. [Version 2] doi: 10.12688/f1000research.3-4.v2 (PMC3976102; doi:10.12688/f1000research.3-4.v2)
Supplement: Peer review history

# Peer review: Referee response for version 2

**Published:** 2014-04-02

**Authors:** Nicholas Feasey

**Affiliations:** 1 Institute of Translational Medicine, University of Liverpool, Liverpool, UK

The authors have responded to the changes I suggested. I have no more to add.

I have read this submission. I believe that I have an appropriate level of expertise to confirm that it is of an acceptable scientific standard.

# Peer review: Referee response for version 1

**Published:** 2014-03-18

**Authors:** Nicholas Feasey

**Affiliations:** 1 Institute of Translational Medicine, University of Liverpool, Liverpool, UK

The authors have reported two cases meliodosis in an area in which this disease was not believed to occur and make an important point about paucity of diagnostic technology in the region.

**Abstract:**

- “ *We report the first two cases of fatal melioidosis in this region...*” The phrasing of this sentence implies that these are the first actual cases of fatal meliod in the region, rather than the first formally diagnosed cases. It currently implies that Meliod is a new/emerging disease to this region rather than a new diagnosis of a condition which is likely to have been present, but unrecognised. 

**Presentation:**

- “ *Presented below are two fatal case reports...*” The case reports were not fatal. "Presented below are two case reports of fatal...” would be better.
   
- The cases are a little brief on detail and if possible, it would be nice to make the corrections/additions suggested by the other two reviewers. Having said that, the weakness of the case descriptions do not detract from the message regarding the description of Meliod in this setting.

**Diagnosis:**

- This is a challenging condition to diagnose and it is important to have a little more detail here – reassuring readers that this really was *B. pseudomallei* is the most important part of this paper. The authors should have described the colonial morphology of the pathogen as *B. pseudomallei* has a characteristic appearance (although I would discourage them from re-culturing it simply to answer this point unless there is a safe laboratory environment to do so). It would also have been useful to record the API20NE score (both the 7 digit number and the percentage ID).What does SMRU stand for? Lastly, what confirmatory test did MORU perform?

**Management:**

- It is a little unusual that management follows the discussion. In particular, the last 2 sentences are conclusions and they should be separated and labelled as such.

I have read this submission. I believe that I have an appropriate level of expertise to confirm that it is of an acceptable scientific standard.

# Peer review: Referee response for version 1

**Published:** 2014-02-17

**Authors:** Blandine Rammaert

**Affiliations:** 1 Service des Maladies Infectieuses et Tropicales, Necker Sick Children's Hospital, Paris, France

F. Nosten and colleagues report two cases of fatal melioidosis in the Myanmar border and remind the international community that this disease was discovered in Myanmar. Melioidosis is probably a forgotten public health issue in Myanmar and this article has the advantage of emphasizing it. However a description of the Myanmar environment, healthcare system and available antibiotics could help to better understand the general background around these two cases.

I have a few comments on the cases:

 

The first case illustrates a chronic pulmonary melioidosis with a fatal septic choc due to *B. pseudomallei*.

- “ *lived in close proximity to the farm*” - Could you please reinterate the context of the Myanmar border; are there irrigated rice fields in the area? Is the patient in close contact with wet soil or any kind of water sources?
   
-  “ *Abdominal examination revealed a 1 cm liver and spleen*” - This statement is not really clear. Do you mean that there are an hepatomegaly and a splenomegaly?
   
-  “ *died more than 8 weeks after the onset of symptoms*” - It would be more helpful to have the time from hospitalization.

 

The second case is about a fatal disseminated melioidosis from cutaneous inoculation. What is less clear is the global clinical presentation. The patient had initial pulmonary oedema. Maybe it would be helpful to discuss pulmonary melioidosis and to have the result of the chest X-ray (if it could have been done in this setting).

- “ *stool sample was noted to be of black color*” - This statement doesn’t bring out any new evidence. 
   
-  “ *A second blood culture obtained one day prior to his death grew Burkholderia pseudomallei after one day of incubation*.” - Does that mean that you got the result after his death? How long did it take to get the result?
   
-  Do the authors think that earlier result of blood cultures could have had a positive impact on the outcome of their two patients? It could be interesting to discuss it.
   
-  Do the authors have any comments on the Ashdown media, which is a cheap, specific and easy-to-make medium. Could it be implemented in the Myanmar border to help diagnosing cases?
   
- Is ceftazidime available in Myanmar?

I have read this submission. I believe that I have an appropriate level of expertise to confirm that it is of an acceptable scientific standard, however I have significant reservations, as outlined above.

# Response

**Published:** 2014-02-24

**Authors:** Cindy Chu

**Affiliations:** Shoklo Malaria Research Unit, Thailand

Thank you for reviewing this case series. Our reply to your comments are as follows:

**Response to comments on the first case:**

1. Land use along the Thailand Myanmar border is primarily agricultural.  Crops that are grown are wide ranging, but most importantly, rice farming is common.  All rice farms have some form of irrigation.  As is custom, this patient and her family lived in a wooden home situated beside the fields of the farm where they worked and would certainly have been exposed to wet soil and natural water sources on a daily basis. 
   
2. “Abdominal examination revealed a 1cm liver and spleen” would mean that both the liver and spleen were enlarged and both measured 1cm. 
   
3. The patient's symptoms had started approximately 8 weeks before admission. She was admitted 3 days prior to her death and the case report describes the events over those 3 days.

**Response to comments on the second case:**

1. At our clinics, situated in a resource-limited setting, we are not able to test stool for occult blood, therefore we rely on the visual examination to determine if there is gastrointestinal bleeding.  In this particular case, the presence of melanic stool would indicate either the severity of his sepsis or an underlying gastrointestinal disorder contributing to his poor clinical condition.
   
2. The second blood culture result grew *B. pseudomallei* within 24 hours.  Results were called immediately to the clinical doctor, however, the patient had already expired the day prior.
   
3. Gram stain, blood culture results, and sensitivities when available are called immediately to the clinical doctor.  Usually, microbiological identification is available within 24 hours of blood culture positivity.  If the first blood culture had been positive then the course of events may have been different.  We agree that that early recognition and confirmation of *B. pseudomallei* by blood culture would save lives.
   
4. Ashdown media would be a suitable selective media for testing a variety of samples for *B. pseudomallei*, particularly from sites with normal flora and if incubated for 48 hrs.  However, it must be noted that when handling *B. pseudomallei *cultures appropriate biosafety level three precautions must be taken, which may be a limiting factor in some areas of Myanmar.
   
5. Ceftazidime is available in hospitals and pharmacies in Myanmar,  however, the fee for service medical system currently present in Myanmar may limit patient access to medical care and treatment.

# Peer review: Referee response for version 1

**Published:** 2014-01-22

**Authors:** Kavitha Saravu

**Affiliations:** 1 Department of Medicine, Kasturba Medical College, Manipal, India

This article certainly raises awareness about the presence of melioidosis in Thai Myanmar border and the expanding map of melioidosis globally. The two cases also reiterate the importance of timely diagnosis and appropriate chemotherapy for successful outcome. This report also underscores the fact that there may be many undiagnosed cases in the region, because of low index of suspicion by the clinicians and lack of facility for diagnosis.

However, a few points could have been included as follows:

 

1. Evidence from a random dextrose level test (432 mg/dL) and a urine dipstick was consistent with anorexia. This statement is not clear. It would have been better if a glycated hemoglobin value was available to rule out diabetes. Additionally, further values of plasma glucose would have been helpful. Also, the finding of the urinary dipstick is not mentioned.
   
2. It is not clear if the patient was treated for vivax malaria.
   
3. The course and events of the patient's hospitalization of 8 weeks has not been mentioned.

I have read this submission. I believe that I have an appropriate level of expertise to confirm that it is of an acceptable scientific standard.
